# A state-of-the-art review on the NRF2 in Hepatitis virus-associated liver cancer

**DOI:** 10.1186/s12964-023-01351-6

**Published:** 2023-11-09

**Authors:** Leila Kalantari, Zahra Rostami Ghotbabadi, Arsalan Gholipour, Hadi Mohammed Ehymayed, Behnam Najafiyan, Parsa Amirlou, Saman Yasamineh, Omid Gholizadeh, Nikoo Emtiazi

**Affiliations:** 1 School of Medicine, Kashan University of Medical Sciences, Kashan, Iran; 2 Shiraz University of Medical Sciences, Shiraz, Iran; 3 Nanotechnology Research Institute, School of Chemical Engineering, Babol Noshirvani University of Technology, Babol, Iran; 4 Department of Medical Laboratory Technics, Al-Noor University College, Nineveh, Iraq; 5 Faculty of Pharmacy, Shiraz University of Medical Science, Shiraz, Iran; 6 Faculty of Medicine, Iran University of Medical Sciences, Tehran, Iran; 7 Free researchers, Biotechnology & Virology, Tehran, Iran; 8 Department of Pathology, Firoozgar Hospital, Iran University of Medical Sciences, Tehran, Iran

**Keywords:** NRF2, Hepatitis virus, Liver cancer, HBV, HCV

## Abstract

According to a paper released and submitted to WHO by IARC scientists, there would be 905,700 new cases of liver cancer diagnosed globally in 2020, with 830,200 deaths expected as a direct result. Hepatitis B virus (HBV) hepatitis C virus (HCV), and hepatitis D virus (HDV) all play critical roles in the pathogenesis of hepatocellular carcinoma (HCC), despite the rising prevalence of HCC due to non-infectious causes. Liver cirrhosis and HCC are devastating consequences of HBV and HCV infections, which are widespread worldwide. Associated with a high mortality rate, these infections cause about 1.3 million deaths annually and are the primary cause of HCC globally. In addition to causing insertional mutations due to viral gene integration, epigenetic alterations and inducing chronic immunological dysfunction are all methods by which these viruses turn hepatocytes into cancerous ones. While expanding our knowledge of the illness, identifying these pathways also give possibilities for novel diagnostic and treatment methods. Nuclear factor erythroid 2-related factor 2 (NRF2) activation is gaining popularity as a treatment option for oxidative stress (OS), inflammation, and metabolic abnormalities. Numerous studies have shown that elevated Nrf2 expression is linked to HCC, providing more evidence that Nrf2 is a critical factor in HCC. This aberrant Nrf2 signaling drives cell proliferation, initiates angiogenesis and invasion, and imparts drug resistance. As a result, this master regulator may be a promising treatment target for HCC. In addition, the activation of Nrf2 is a common viral effect that contributes to the pathogenesis, development, and chronicity of virus infection. However, certain viruses suppress Nrf2 activity, which is helpful to the virus in maintaining cellular homeostasis. In this paper, we discussed the influence of Nrf2 deregulation on the viral life cycle and the pathogenesis associated with HBV and HCV. We summed up the mechanisms for the modulation of Nrf2 that are deregulated by these viruses. Moreover, we describe the molecular mechanism by which Nrf2 is modulated in liver cancer, liver cancer stem cells (LCSCs), and liver cancer caused by HBV and HCV.

Video Abstract

**Supplementary Information:**

The online version contains supplementary material available at 10.1186/s12964-023-01351-6.

## Introduction

The 2020 World Cancer Data Report estimates that each year, there are around 906,000 new cases and 830,000 deaths due to liver cancer, and these numbers are continually rising. The most common form of liver cancer, hepatocellular carcinoma (HCC), is now the cause of cancer deaths worldwide [1]. There are currently no definitive therapies for liver cancer, despite the fact that it is treated with organ transplantation, surgical resection, and anticancer medications. This is primarily due to a lack of donor livers and tumor heterogeneity [2]. Additionally, because of the disease’s fast development, many patients who sought therapy missed the chance for the first surgical resection. When there is a big tumor volume or a significant tumor load, direct resection may result in severe trauma. In contrast, a lack of appropriate remaining liver capacity may result in postoperative liver failure [3]. Intertumoral heterogeneity is observed in etiologies, mutational landscapes, transcriptomes, and histological representations of HCC and cholangiocarcinoma (CCA), two types of liver cancer [4, 5]. HCC is thought to originate and advance mostly as a result of infectious causes, such as the hepatitis B virus (HBV), hepatitis C virus (HCV), and hepatitis D virus (HDV) [6]. Approximately 257 million individuals worldwide are thought to have HBV infection, and 71 million have HCV infection. Therefore, effective interventions for those with HBV or HCV infections are required to lower liver cancer mortality rates [7]. Through several methods, such as insertional mutations brought on by viral gene integration, epigenetic alterations, and the production of chronic immunological failure, these pathogens drive hepatocyte metamorphosis. Additionally, the study and identification of chronic hepatitis E virus (HEV) infection during the last 10 years suggest that this common hepatitis virus may potentially be able to cause HCC [8, 9].

**Fig. 1:**
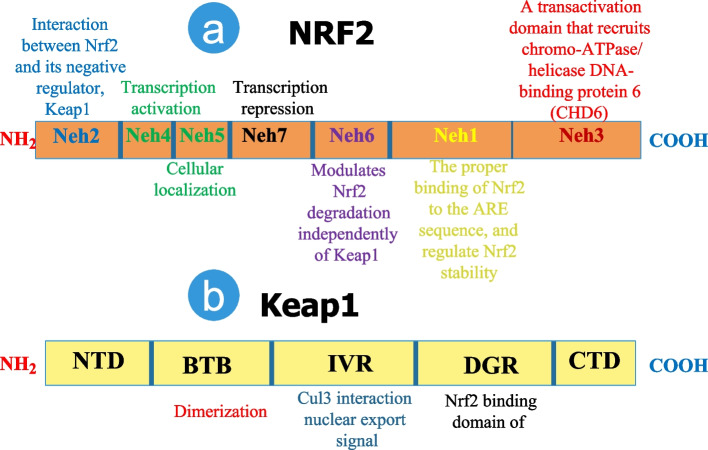
Major structures and functions of the Nrf2 (a) and Keap1 (b) domains are shown on a schematic diagram. 605 amino acids comprise the protein Nrf2, and its stability and transcriptional activity are regulated by seven functional domains called Nrf2-ECH homology (Neh) 1-7. Keap1, the primary intracellular regulator of Nrf2, is a 624-amino-acid protein composed of five domains: an N-terminal domain (NTD), a broad complex, tramtrack and bric-a-brac (BTB) domain, an intervening region (IVR), a double-glycine repeats (DGR) domain, and a C-terminal domain (CTD). Each of these domains plays a critical role in inhibiting Nrf2 activity. The DLG and ETGE motifs are significant for Keap1's binding to the N-terminal Neh2 domain of Nrf2. In response to oxidative stresses, the DLG motif in Nrf2 is uncoupled from the DGR domain in Keap1, protecting the protein from ubiquitination and degradation [20–22]

Nuclear factor E2-related factor 2 (NRF2), alternatively referred to as nuclear factor (erythroid-derived 2)-like 2 (NFE2L2), is a member of the Cap‘n’Collar/basic leucine zipper (CNC-bZIP) family of transcription factors. The NRF2 protein is of great importance in the regulation of redox homeostasis through its ability to stimulate the transcription of numerous genes that are involved in antioxidant defense mechanisms [10]. Because its cytoprotective actions are believed to constitute the primary cellular defense mechanism against exogenous and endogenous insults, including xenobiotics and oxidative stress (OS), NRF2 has historically been considered a tumor suppressor [11]. NRF2 plays a significant role in oncogenic and cytoprotective pathways inside cancer cells, exerting direct or indirect influence on carcinogenesis, proliferation, apoptosis, redox balance, metastasis development, and resistance to anticancer treatments [12]. NRF2 has both tumor suppressor and oncogenic roles in cancer, helping to keep cancer from progressing and allowing cancer cells hang on. In many malignancies, its expression is heightened, and it is linked to tumor aggressiveness, progression, and therapeutic resistance [13]. Deregulation of Nrf2 is an essential component in oncogenesis and is seen in various cancers. Thus, increased NRF2 activity has far-reaching consequences for the phenotype of tumor cells, including radio/chemoresistance, apoptosis protection, invasiveness, antisenescence, autophagy insufficiency, and angiogenesis. Both direct epigenetic and genetic modifications to NRF2 control and the complicated interaction of NRF2 with a plethora of oncogenic signaling pathways may lead to NRF2 dysregulation. Changes in the cellular environment, such as those seen during inflammation, also have a role in the deregulation and sustained activation of NRF2. As a result, there are a wide variety of methods that may be used to determine if NRF2 is anti- or protumorigenic. To effectively use NRF2 as either an activation or inhibition target in chemoprevention or cancer treatment, a thorough comprehension of these approaches is crucial [14]. Additionally, a number of research on chemopreventive medications have hypothesized that the activation of the transcription factor NRF2 is a crucial mechanism by which these treatments’ favorable effects on the prevention of carcinogenesis and several other chronic illnesses are achieved [15]. Recent genetic investigations of human tumors have shown that NRF2 may, on the other hand, be carcinogenic and contribute to treatment resistance. Therefore, it is debatable whether activating NRF2 or, conversely, inhibiting it is a beneficial method for cancer therapy or prevention [16]. As a result of their interaction with the Keap1 (Kelch-like ECH-associated protein 1) is an adaptor subunit of Cullin 3-based E3 ubiquitin ligase (KEAP1-E3) complex and subsequent ubiquitination and proteasomal degradation, NRF2 proteins usually are kept at deficient levels [17]. However, the KEAP1-NRF2 connection is broken by oxidative and/or electrophilic stressors, which causes NRF2 to assemble and become transactivated [18]. NRF2 is commonly activated in various cancer types via various mechanisms in recent years, including genetic alterations in the KEAP1-NRF2 pathway, according to a growing body of research. This showed that NRF2 suppression is an effective method for treating cancer [19] (Fig. 1).

The continuous overproduction of viral proteins can lead to an elevated concentration of reactive oxygen species (ROS) and free radicals. Moreover, it is noteworthy that the activation of NRF2 exhibits a wide range of antiviral capabilities, which is unexpected given its regulatory impact on interferon (IFN) [23]. These capabilities encompass the inhibition of viral replication in severe acute respiratory syndrome-Corona Virus (SARS-CoV2), Zika virus, and Herpes Simplex virus infections in vitro [24–26]. However, NRF2 activation seems pro-viral in certain circumstances, presumably because it promotes cell survival, which suggests that some viruses have adapted to use NRF2 to aid in reproduction. Recent lines of evidence support the idea that, in general, NRF2 activation is protective during viral infection. At the same time, there are occasions where certain species of virus hijack NRF2 as an integral element of replication. The defense may be mediated by antiviral actions, preventing virus reproduction, or by preventing cells from dying, preventing tissue harm [27].

Most forms of liver illness may be traced back to OS, including drug-induced liver damage, viral hepatitis, and alcoholic hepatitis. To combat OS, cells and organisms rely heavily on the Keap1-Nrf2 system. To further facilitate fatty liver disease in mice, numerous natural Nrf2 activators can control lipid metabolism and OS of liver cells [28, 29]. The upregulation of hemeoxygenase-1 by elevated Nrf2 in viral hepatitis serves to suppress HCV replication. Mice with autoimmune liver disease benefit greatly from elevated Nrf2 because it helps them avoid liver damage. By lowering ROS levels, increased Nrf2 prevents liver fibrosis in cirrhotic livers [30]. In the development of liver cancer, Nrf2 has a dual role [31]. At this time, a drug that acts as a Nrf2 agonist is available for human use. As a result, treating liver disorders may include stimulating the Nrf2 pathway to stimulate the production of cytoprotective genes. In this study, researchers provide a synopsis of the literature on the links between OS and liver damage, as well as the essential function of the Nrf2 pathway in a wide range of liver illnesses [32]. In addition, to accumulate the necessary number of mutations required to turn them into HCC progenitor cells (HcPC), hepatocytes exposed to OS and within which oncogenic mutations accumulate must be able to tolerate persistent ROS buildup and continue to multiply under such circumstances. NRF2 is, without question, the most critical factor in this procedure. Short-term activation of NRF2 may be beneficial, while long-term activation due to p62 buildup is oncogenic. To prevent chronic liver inflammation from leading to HCC, the use of particular NRF2 inhibitors may be preferable to the use of antioxidants and NRF2 activators as cancer-preventive drugs [33]. It was also shown that during HBV infection, Nrf2, which functions as a liver regeneration and antioxidative protein, regulates the enhancement of liver regeneration, hence linking OS to hepatic regeneration and ensuring the survival of injured cells [34, 35]. This article summarizes the pathways involved in the modulation of Nrf2, which are disrupted by HBV and HCV. Additionally, the paper discusses the consequences of Nrf2 deregulation on the life cycle of these viruses and the associated pathogenesis. Furthermore, we elucidate the molecular mechanism underlying the regulation of Nrf2 in hepatocellular carcinoma, hepatic cancer stem cells (CSCs), and liver cancer associated with HBV and HCV.

## NRF2 in cancer

Ionizing radiation, prooxidants, hypoxia, nutritional deprivation, iron deficiency, viral infection, lipid excess, metabolic stress, and proteotoxic aggregates are only a few of the many exogenous and endogenous stressors that may trigger the generation of ROS. ROS production may be triggered by metabolic abnormalities or an oxygen supply/demand imbalance. While ROS at low and moderate levels may play a signaling role in normal cellular function, ROS at high levels promote tumorigenesis by generating DNA mutations and oncogenic transformation. It is worth mentioning that cancer cells frequently exhibit elevated levels of cellular glutathione (GSH) and ROS [36, 37]. This is especially true for chemo-resistant cancer cells, were increased ROS levels trigger antioxidant defense mechanisms, such as NRF2. These mechanisms contribute to the development of chemoresistance by altering metabolic processes and mitigating drug-induced oxidative stress, which typically results in the demise of chemo-sensitive cancer cells [38, 39]. Somatic mutations and other abnormalities in the Nrf2 genes, as well as well-known tumor suppressor genes such as tumor protein p53 (TP53), cyclin-dependent kinase inhibitor 2 A (CDKN2A), Phosphatase and tensin homolog (PTEN), and phosphatidylinositol-4,5-bisphosphate 3-kinase catalytic subunit alpha (PIK3CA), have been identified in various malignancies. Tumor cells exhibit somatic mutations in the Nrf2 genes, along with other mechanisms that impact Nrf2 binding, resulting in the generation of abnormal Nrf2 activation. The unregulated activation of Nrf2 has been observed to confer resistance to antineoplastic drugs and ROS in tumor cells while also influencing their metabolic reprogramming [31]. Recent research has shown that NRF2 activation regulates autophagy, unfolded protein response (UPR), micropinocytosis, and metabolic reprogramming, all contributing to cancer cell survival and treatment resistance [38]. Chemical carcinogens cannot cause cancer if NRF2 is activated in normal cells in a controlled manner. High NFE2L2/NRF2 expression, however, increases tumor growth, metastasis, and resistance to anticancer therapy, as shown by recent research. This oncogenic activity of NRF2 is primarily attributable to the somatic mutation of KEAP1. In the clinic, high levels of NRF2 expression in tumors almost always indicate a dismal prognosis. In order to create novel therapeutic options for the efficient treatment of cancer, it is crucial to comprehend the molecular pathways through which NRF2 aids cancer cells in evading cell death [40]. The antioxidant effects of NRF2 are just one of the ways in which its overexpression promotes cancer cell proliferation and survival. Other mechanisms include the upregulation of anabolic metabolism and chemoresistance via the upregulation of phase II enzymes and drug efflux transporters [41].

### NRF2 in liver cancer

Stabilizing Nrf2 in the cytoplasm and then moving it into the nucleus to increase the transcription of cytoprotective target genes is an intrinsic and adaptive OS mechanism. When activated, p62 may sequester Keap1 in autophagosomes, transport Nrf2 to the nucleus, and control anti-OS genes. p62 is the upstream component of the P62-Keap1-Nrf2 pathway. The Nrf2-related signaling system protects HCC cells against ferroptosis, mediating cellular resistance to sorafenib. Therefore, inducing ferroptosis relies heavily on the p62-Keap1-Nrf2 pathway. The expression of Nrf2 is linked to medication resistance and a poor prognosis, and its upregulation is common in HCC tissues. The tumor-killing effects of sorafenib in HCC were amplified by metformin, which induced ferroptosis by blocking an Nrf2-related pathway. The combination of sorafenib and metformin inhibited the growth of HCC cells through the p62-Keap1-Nrf2/HO1 signaling pathway, as shown by the findings of the Nrf2 knockdown and p62 knockdown studies [42]. Cell proliferation and cell cycle progression mediated by microcystin-LR (MC-LR) were suppressed by silencing Nrf2. Taken together, researcher’s findings support a beneficial function for Nrf2 in carcinogenesis and imply that MC-LR-induced overexpression of Nrf2 in cancer cells stimulates the development of liver cancer cells [43].

High expression/activation of hypoxia-inducible factor-1 (HIF-1α) is a hallmark of hypoxic tumors like HCC. NRF2, another essential transcription factor, is likewise overactive in a constitutive manner in HCC. NRF2 knockdown significantly decreased the nucleus accumulation of HIF-1α without changing its mRNA expression, while HIF-1α silencing did not affect NRF2 protein expression in HepG2 human hepatoma cells. NRF2 expression was upregulated with HIF-1α overexpression in diethyl nitrosamine-induced hepatocarcinogenesis in wild-type (WT) mice. However, with Nrf2 knockout mice, this was no longer the case. Tumour samples from patients with HCC also showed upregulation of NRF2 and HIF-1, as well as evidence of their interaction. Under normal oxygen conditions, HIF-1α is hydroxylated by a protein with a HIF-prolyl hydroxylase domain (PHD), promoting its subsequent ubiquitination and proteasomal destruction. By directly binding to HIF-1α’s oxygen-dependent degradation (ODD) domain, NRF2 contributes to pseudohypoxia by inhibiting PHD2-mediated hydroxylation, concurrent von-Hippel-Lindau recruitment, and ubiquitination of HIF-1α [44]. Although selenium’s anticancer effects in vitro are promising, their translation to the clinic has been less than stellar due to the poor oxygenation of solid tumors. Oxygen therapy, according to a hypothesis by Cheng Wang et al., may increase the therapeutic effectiveness of selenium in hypoxic tumors by altering their redox environment [45]. Researchers discovered that hyperoxia, in contrast to hypoxia, facilitated the rapid oxidation of hydrogen selenide (H2Se), produced by the selenium metabolism in cells, to generate H2O2. This, in turn, inhibited the expression level of Nrf2, increased the phosphorylation of p38 and MKK4, and sped up the apoptosis and autophagy processes. Once the Nrf2 gene was silenced, apoptosis was exacerbated by the addition of hyperoxia and the pro-oxidant selenium compounds. These results show that oxygen may considerably boost the anti-HCC impact of selenium compounds by controlling the Nrf2 and MAPK signaling pathways, opening the door to selenium’s use in clinical therapy for hypoxic tumors [45].

Ablation of p62 in hepatocytes, utilizing Moscat et al.‘s p62F/F mice, entirely suppressed HCC formation in Tsc1hep animals and markedly attenuated it in MUP-uPA mice given a high-fat diet and STZ-HFD mice. Lack of p62 was shown to have a substantial impact on the mTORC1 pathway, which in turn downregulated the production of c-Myc and the NRF2-activated antioxidant response. Numerous genes whose products are involved in glutathione production and protection of cells from OS were downregulated with the inhibition of NRF2 function. Using p62F/F animals created by Moscat et al., hepatocyte-specific p62 ablation entirely prevented HCC formation in Tsc1-hep mice. It significantly slowed it down in STZ-HFD mice and MUP-uPA mice fed on HFD. The mammalian target of rapamycin complex 1 (mTORC1) pathway, whose downregulation greatly affected the expression of c-Myc and the NRF2-activated antioxidant response, was identified to be the main oncogenic signaling route that is impacted by the lack of p62. Numerous genes whose products are involved in glutathione production and protecting cells from OS were downregulated together with the inhibition of NRF2 activity [33]. The absence of protein kinase C (PKC) λ/ι in hepatocytes enhances autophagy and oxidative phosphorylation, according to research. ROS are produced as a consequence, and NRF2 uses both cell-autonomous and non-autonomous processes to promote the development of HCC. Although PKCλ/ι promotes carcinogenesis in cancer models driven by oncogenes, new research shows that it acts as a tumor suppressor in more intricate carcinogenic pathways. PKCλ/ι levels consistently correlate negatively with the grade of the HCC histological tumor, designating this kinase as a tumor suppressor in liver cancer [46].

### NRF2 in liver cancer stem cells

Similar to how genetic factors do, nongenetic elements hierarchically organize tumor tissues, with a subset of self-renewing cancer stem cells (CSCs) supporting the long-term clonal maintenance of the tumor [47]. The CSC hypothesis was put out forty years ago and claims that a small number of specialized stem cells drive tumor development like how healthy tissues regenerate [48]. As with their healthy counterparts, distinct anatomical areas harboring CSCs have been found inside the microenvironment of different tumors. These anatomical regions are known as “niches.“ Numerous studies have so far shown that CSC niches play a role in the upkeep, control of renewal, differentiation, and adaptability of CSCs [49].

Liver cancer stem cells (LCSCs) impact HCC carcinogenesis, development, and therapy resistance. Several CSC markers, such as CD133, CD90, CD44, the oval cell marker OV6, EpCAM, CD13, CD24, DLK1, 21, ICAM-1, CD47, Lgr5, and keratin19, have been discovered in liver cancer, indicating the presence of a small proportion of cancer cells with stem cell traits (self-renewal and differentiation) [50, 51]. Cancer development and progression may be driven by LCSCs, a subset of tumor cells. Given their importance, scientists are looking for biomarkers that may be utilized to identify or control LCSCs as potential therapeutic targets in the diagnosis and treatment of chronic liver disease and HCC [52, 53].

Multiple studies show that many viruses contribute to cancer’s aggressiveness by facilitating the development of CSC characteristics [54–56]. Chronic viral infection (such as HBV and HCV), alcohol, and non-alcoholic fatty liver disease all produce a prolonged inflammatory/regeneration process, leading to many different kinds of liver cancer. Liver cancer is initiated and/or promoted by the continued growth of stem/progenitor cells, the accumulation of genetic and/or epigenetic alterations, and the microenvironment modification. In addition, this method may help hepatic stem/progenitor cells develop into LCSCs [50, 57]. The induction of CSC formation, activation of stemness-related factors, and tumorigenicity by HBV non-structure protein X (HBx) were all mitigated through the overexpression of miR-124 or the silencing of lncRNA-MALAT1 [58]. Using the farnesoid X receptor (FXR) route and drug metabolism as examples, Ng et al. found that C-terminal-shortened HBx enhances HCC carcinogenesis by inducing CD133 LCSCs. Spheroids, expression of epithelial-mesenchymaltransition (EMT) and CSC markers, and tumor development in immunodeficient mice are all significantly boosted by HCV infection of converted human hepatocytes. HCV infection in conjunction with alcohol or a high-fat diet induces LCSC production and carcinogenesis, as was recently shown by Chen, Kumar, and colleagues through Toll-like receptor 4-NANOG (TLR4-NANOG) signaling [59–62].

**Fig. 2:**
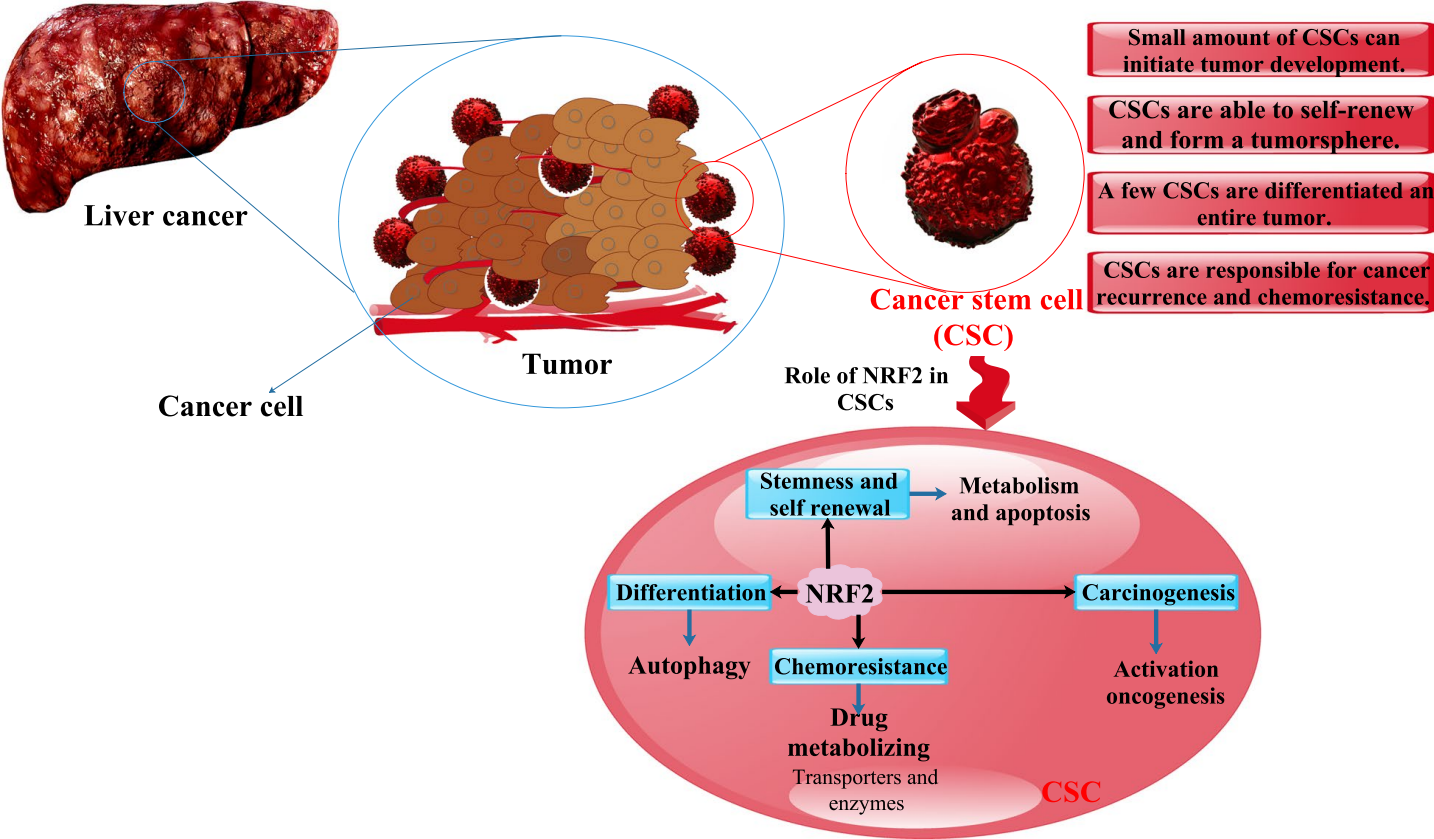
Liver CSC properties and NRF2's function in the liver. By regulating downstream enzymes and transporter members, Nrf2 enhances drug metabolism and drug resistance, triggers a DNA damage response, and plays a role in the development of specific stem cells. Here, we summarize the research on NRF2 and its impact on CSCs' stemness, self-renewal capacity, tumorigenesis, and chemoresistance [67]

The relevance of ROS signaling to tumorigenicity, tumor development, and relapse has recently begun to be investigated. In an oxidizing tumor microenvironment, CSCs are more likely to be maintained and survive if they express higher levels of the transcription factor NRF2, a master regulator of antioxidant gene expression. The multidrug resistance of CSCs may also be facilitated by NRF2-mediated upregulation of ATP-binding cassette (ABC) transporters, especially the CSC marker breast cancer resistance protein (BCRP). These results, in conjunction with the growing body of data demonstrating aberrant KEAP1-NRF2 signaling in cancer cells, point to a hitherto unrecognized function for NRF2 in CSC maintenance and survival [63]. Additionally, NRF2’s location at the nexus of cellular metabolism and redox homeostasis raises the possibility that it plays a significant role in the biology of cancer cells and CSCs. NRF2 controls the expression of enzymes involved in redox homeostasis, cell metabolism, and xenobiotics detoxification to promote survival in normal cells, cancer cells, and CSCs. CSCs as a result of the adaptive response triggered in response to unfavorable conditions present in the microenvironment or brought on by therapeutic interventions [63]. For example, CSCs increase cell flexibility and survival by swiftly alternating between oxidative phosphorylation (OXPHOS) and glycolysis, depending on the situation. Although further investigation is required to determine the precise involvement of this transcription factor in the metabolic reprogramming of CSCs, such as the redirection of glucose and glutamine towards the pentose phosphate pathway as observed in typical cancer cells, NRF2 seems to operate downstream primarily. Its primary function appears to be the regulation of redox regulators, thereby influencing the levels of ROS within the cell. The reliance on NRF2 signaling potentially suggests a susceptibility of CSCs that could be targeted through therapeutic interventions. However, additional investigation is necessary to ascertain the extent to which NRF2 can regulate crucial metabolic pathways of CSCs [64]. An association between CSCs and NRF2 activation is also suggested by the flow cytometry-based separation of CSCs. According to proteomic research, CD44 variation 9 (CD44v9) HCC CSCs express much more NRF2 than CD44v9- HCC CSCs. We conclude that CD44v9 is a predictive marker in HCV(+) HCC patients linked with Nrf2-mediated resistance to OS and a possible biomarker of tumor-initiating stem-like cells [65]. The NRF2 pathway was shown to be significantly involved in the survival, maintenance, and therapeutic resistance of CSCs, as demonstrated by these findings. The autophagy adaptor protein p62 (encoded by the SQSTM1 gene) has been hypothesized to function as an oncoprotein. Selective p62 inhibition reduced the ability of acute myeloid leukemia cells to initiate malignancy, suggesting a function for p62 in CSCs. Additionally, several studies have linked the upregulation of NRF2 in CSCs to p62. It was discovered that HCC-initiated cell survival and growth depended on p62 accumulation, which boosted NRF2 activity [66]. While researchers have delved into this functional crosstalk to some level in cancer cells, their exploration of LCSCs is only getting started (Fig. 2).

## NRF2 in viral infection

**Fig. 3:**
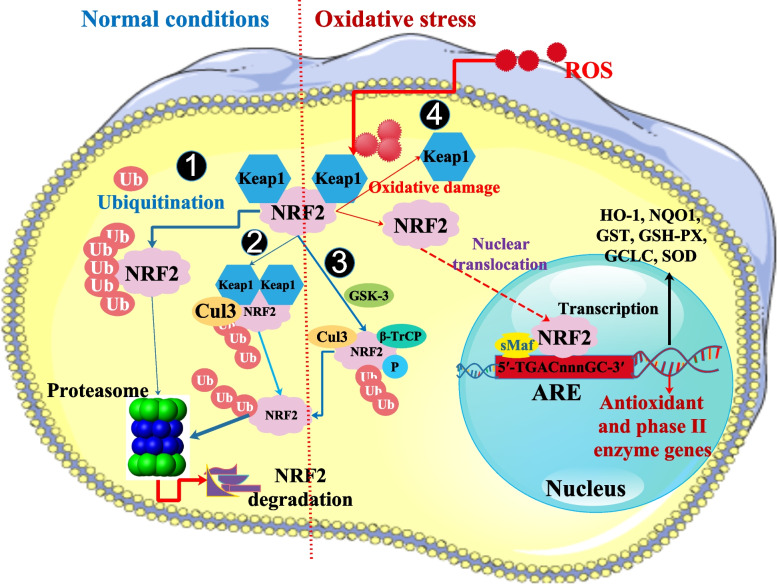
Keap1/Nrf2 signaling's depiction of its regulatory processes. A negative effect on Nrf2, Nrf1, and Nrf3 expression. In standard settings, proteasome activity keeps Nrf2 expression at a modest level. Keap1 binds to and sequesters Nrf2 in the cytosol, which is then ubiquitinated by the Keap1-Cul3-Ring-box1 protein (Rbx1, also called Roc1) complex and then degraded by the proteasome. Glycogen synthase kinase 3 (GSK-3) inhibits Nrf2 function and may promote the degradation of Nrf2. To recruit the E3 ligase adapter-TrCP, GSK-3 phosphorylates Nrf2. The ubiquitin-proteasome degradation of Nrf2 is triggered by GSK-3/-TrCP, although it does not need Keap1. 4) Positive regulation of Nrf2: when ROS levels are high, Nrf2 is unbound from Keap1 and moves to the nucleus, where it activates antioxidant genes, including Heme oxygenase 1 (HO1) and NAD(P)H dehydrogenase (Quinone) 1 (NQO1). In response to oxidative stress, Nrf2 dissociates from Keap1 and moves to the nucleus, which associates with sMaf to form a heterodimer. By binding to ARE sequences, heterodimeric Nrf2-sMaf transcription factors stimulate the production of antioxidant genes. Instead, cytoprotective gene expression is suppressed when Nrf2 is phosphorylated by GSK-3 because -TrCP facilitates Nrf2's association with a Cul1 ubiquitin ligase complex (Skp1-Cul1-Rbx1/Roc1) [20, 74]

Commonly, Nrf2 is found in the cytoplasm and mainly binds to keap1. The transcription of cytoprotective genes is induced in response to oxidative and electrophile stress, however, because Nrf2 moves to the nucleus and binds to antioxidant response elements (ARE) [68]. These genes encompass a range of functional categories, including antioxidant enzymes such as glutamate-cysteine ligase (GCL), drug-metabolizing enzymes like cytochrome P450s and glutathione S-transferases (GSTs), molecular chaperones, DNA repair enzymes, and proteasome subunits [69]. The expression of Nrf2-regulated genes is contingent upon the formation of Nrf2 heterodimers with small Maf proteins (MafG, MafK, MafF), a process necessary for effective binding to the ARE/EpRE (antioxidant response element/electrophilic response element). The cell can maintain the redox equilibrium and remove proteins harmed by oxidative and xenobiotic stress by transcribing these protective genes [70]. Most viruses lead to OS and boost radical and ROS activity, which triggers the cellular defense mechanism to activate the Nrf2 and increase the production of cytoprotective genes [71]. However, there are situations in which Nrf2 activation does not rely on ROS and instead occurs directly via the ROS-independent route. The activation of Nrf2 is a common viral effect that plays a role in virus pathogenesis, infection progression, and even chronicity. While this pathway is essential for cellular homeostasis, certain viruses employ their ability to suppress Nrf2 activation to their advantage [72]. Furthermore, the activation of Nrf2 has been shown to augment the activity of the innate immune system in the process of attenuating or eradicating various bacterial and viral pathogens [73] (Fig. 3).

The release of this vital transcription factor causes it to go into the nucleus, which stimulates the production of antioxidant enzyme (AOE) genes. The AOEs prevent oxidative damage from ROS and reactive nitrogen species (RNS). However, viral infections may gradually reduce AOE expression by suppressing NRF2 activation or boosting its degradation, resulting in the buildup of reactive species, cellular damage, and lung injury [75]. Heme oxygenase 1 (HO1) is a significant anti-inflammatory target of NRF2, and NAD(P)H dehydrogenase (Quinone) 1 (NQO1) is an essential enzyme involved in the detoxification of quinones and redox signaling [76]. Among AOE, glucose-6-phosphate dehydrogenase (G6PD) is necessary to maintain the cytosolic pool of NADPH and, therefore, the cellular redox balance. NRF2 interacts with many innate immune signaling molecules to inhibit the expression of pro-inflammatory genes, restrict the IFN response, and minimize inflammation and tissue damage in addition to transcriptionally activating AOE [77, 78]. The activation of Nrf2 has two effects: it may both promote and prevent the development of viral diseases. However, in general, during viral infection, Nrf2 activity protects host cells. Activation of Nrf2 has been found to suppress Zika virus, Ebola virus, and influenza A virus in cellular assays [27, 79]. It has also been proven in vivo animal research to decrease mortality and increase life expectancy following viral infection. This shows that various viruses are still susceptible to the antiviral effects of Nrf2 activation. Since these antiviral effects are preserved, it is likely that Nrf2 regulates a broad-acting antiviral program and potentially be a therapeutic target during viral infections [80, 81].

## NRF2 in hepatitis virus

Multiple studies show that HCV has opposing effects on the gene expression of radical-producing and detoxifying systems in HCV-positive cells, with the former being stimulated by HCV and the latter being inhibited by the virus. Increased levels of reactive oxygen species have been linked to insulin resistance, promoting the progression of fibrosis, cirrhosis, and HCC [9]. The induction of autophagy is essential for the HCV life cycle, and this occurs in part because of the high ROS level [82]. In comparison, the HBV issue seems considerably more convoluted. The cellular immune response and viral regulatory proteins may have an impact, for example, by disrupting mitochondrial function and raising intracellular ROS levels [83]. However, some research suggests that HBV-positive cells may boost their production of cytoprotective enzymes by activating the Nrf2/ARE-dependent gene expression [84]. To avoid being eliminated by the immune system, HBV-positive cells may start the Nrf2/ARE system as a means of escaping detection. The balance between ROS-inducing and ROS-inactivating pathways may shift throughout time, from acute infection to chronic infection to HCC. There may be points where enhanced Nrf2-activation hinders viral eradication. HBV-associated HCC might resist treatment if Nrf2 activity is high [85].

### NRF2 in hepatitis C virus

**Fig. 4:**
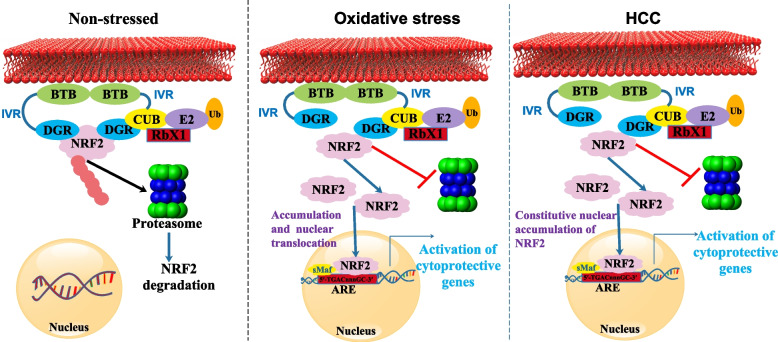
Non-stressed, OS, and HCC cells all use the Keap1-Nrf2-ARE pathway. Keap1 is distinguished by its tramtrack and bric-a-brac (BTB) domain, an intervening region (IVR), and double-glycine repeats (DGR) domains. Keap1 increases Nrf2 breakdown in the non-stressed state, keeping Nrf2 levels low. To keep redox homeostasis stable throughout OS, Nrf2 is active. Prolonged activation of Nrf2 in HCC causes cells to multiply and develop treatment resistance

The core, E1, E2, NS4B, and NS5A proteins of HCV were shown to activate the antioxidant defense Nrf2/ARE pathway in a study. In reaction to ROS buildup, all five proteins activated protein kinase C (PKC), activating Nrf2. Further, ROS-independent activation of Nrf2 was achieved by expression of core, E1, E2, NS4B, and NS5A proteins. Core and NS5A’s effects were mediated by casein kinase 2 (CK2) and phosphoinositide-3 kinase, whereas NS4B, E1, and E2’s effects were not mediated by PKC, CK2, phosphoinositide 3-kinase (PI3K), p38, or extracellular signal-regulated kinase (ERK). Overall, HCV proteins generated a robust up-regulation of the antioxidant defense system at the earliest stage of expression [86]. The autophagic adaptor protein p62 is phosphorylated on Ser349 (pS349 p62) in response to elevated levels of ROS, initiating autophagy. Therefore, Nrf2 is dissociated from the Keap1 complex because pS349 p62 attaches to Keap1 with increased affinity. Despite the presence of increased quantities of pS349 p62, no activation of cytoprotective genes takes place in HCV-positive cells despite the released Nrf2 being expected to stimulate as a heterodimer with the sMaf proteins the expression of Nrf2/ARE-dependent genes. Free Nrf2 is inhibited from entering the nucleus in HCV-positive cells because delocalized sMaf proteins are positioned at the replicon complexes on the cytoplasmic face of the endoplasmic reticulum (ER). Reducing ROS production reduces pS349 p62 and hinders autophagy induction. When autophagy is blocked, or ROS is scavenged, fewer virus particles are shed into the environment. As a whole, our findings reveal a complex mechanism by which HCV counteracts pS349 p62-induced activation of Nrf2 by suppressing Nrf2/ARE-mediated gene expression. This keeps ROS levels high, which triggers autophagy and promotes HCV particle release [87] (Fig. 4).

Hepatocytes develop oxidative stress after exposure to toxic compounds or carcinogenic agents, which increases the nuclear import of the transcription factor Nrf2. Increased levels of Nrf2 expression under OS state control tumor cell proliferation, survival, and invasion. Through downregulating the apoptotic signaling pathway, Zhang et al. found that Nrf2 aided HCC growth and metastasis. To produce OS indicators, HCV infections may downregulate the expression of Nrf2-ARE-regulated genes. In addition, HCV-induced hepatotoxicity led to a time-dependent rise in Nrf2 accumulation, which in turn triggered the transcriptional activation of Nrf2-ARE-related genes involved in the expression of PI3K, CK2 and mitogen-activated protein kinases [88, 89].

### NRF2 in hepatitis B virus

In vitro and in vivo, human HBV strongly activates the genes controlled by the Nrf2/ARE protein. Through c-Raf and mitogen-activated protein kinase kinase (MEK), the HBV-regulatory proteins (HBx and HBV large surface protein (LHBs)) cause this. Compared to control cells, HBV-positive cells are better protected against oxidative damage because of the Nrf2/ARE-mediated activation of cytoprotective genes by HBV. Additionally, HBV-positive cells have a markedly elevated expression of the Nrf2/ARE-regulated proteasomal component proteasome subunit beta type-5 (PSMB5), which is connected to a lower level of the immunoproteasome subunit PSMB5i. According to this observation, even after receiving IFNα/γ therapy, HBV-positive cells exhibit increased constitutive proteasome activity and reduced immunoproteasome activity compared to control cells. The survival of the infected cell may be ensured by the HBV-dependent stimulation of Nrf2/ARE-regulated genes, which may also help to influence the immunological response to HBV and hence aid in the spread of infection [84].

Hepatitis B surface antigen (HBeAg) secretion is absent, and HBsAg secretion is deficient in HBV genotype G (HBV/G). Hepatoma cells that expressed HBV/G and HBV/A as well as infected HepaRG cells were examined. In HBV/G replicating cells, the release of viral particles is unaffected; however, the secretion of subviral particles, despite their high production levels, is compromised. The ER collects these subviral particles, which have an elevated density and a mostly filamentous shape. The PreS1PreS2 domain of HBV/G, which aggregates, blocks HBsAg-secretion in the ER and reduces LHBs’ capacity to serve as transcriptional activators. An increased number of ROIs is caused by intracellular HBsAg buildup and poor Nrf2 cytoprotective transcription factor activation. As a result, c-Jun N-terminal kinase (JNK) is activated, and as a result, Insulin receptor substrate 1 (IRS-1) is Ser-phosphorylated. Insulin signaling, a crucial component of liver regeneration, is known to be impaired as a result. The ability to inactivate reactive oxygen intermediates (ROIs) declines along with a reduction in HBV-induced Nrf2 activation. There may be a connection between this and genotype-specific pathophysiology [90].

**Fig. 5:**
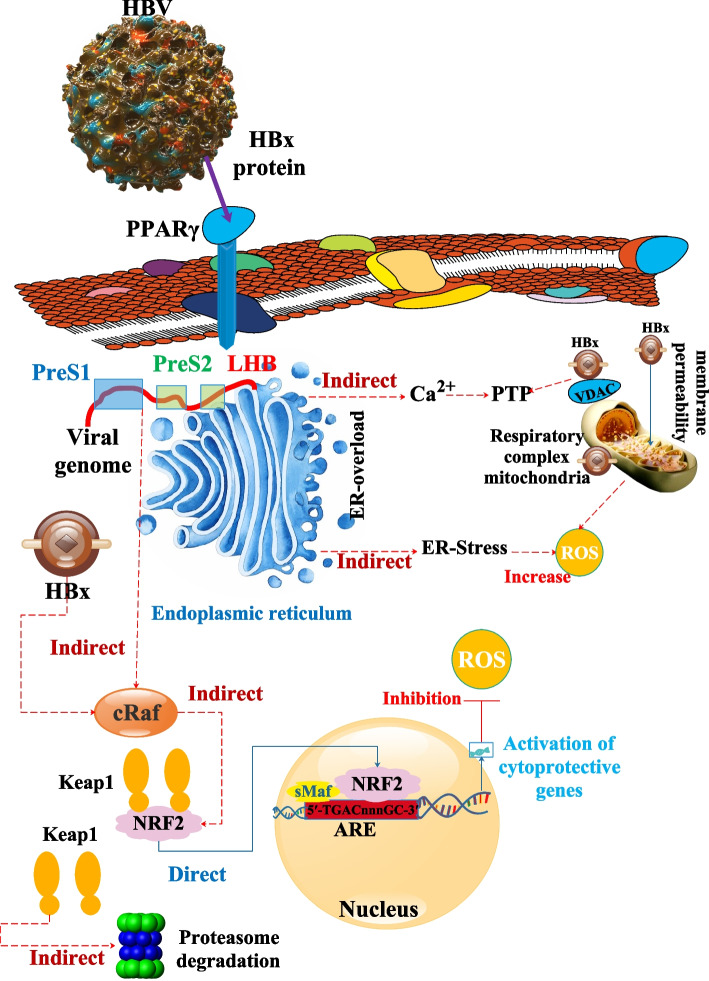
ROS activation and deactivation as a result of HBV. It has been reported that HBx disrupts the integrity of the outer mitochondrial membrane via interactions with that membrane. Furthermore, HBx binds to Voltage-dependent anion-selective channel protein 3 (VDAC3), modulating the permeability transition pore (PTP). The effect of HBx on PTP function was reported as an increase in cytoplasmic and mitochondrial Ca2+ concentrations. Inhibiting the expression of subunits of respiratory complexes I, II, IV, and V, HBx reduces the activity of the respiratory chain complex. A loss of mitochondrial membrane potential is the last stage in this chain of events, which might generate ROS. LHBs are retained and accumulated in the ER after overexpression, which is linked to ER stress and the activation of the UPR. Ca2+ release in the cytoplasm due to the UPR may initiate ROS production. Transcriptional activators include HBx and LHBs (if the PreS2-domain faces the cytoplasm). The activation of c-Raf, which was reported as essential for the HBV-dependent activation of Nrf2, is brought on by both regulatory proteins. Cytoprotective genes with an ARE sequence in their promoter begin to express with the HBV-dependent activation of Nrf2. ROS detoxification is made possible by the increased production of cytoprotective genes in HBV-expressing cells [85]

In addition to superinfecting persons by infecting those with chronic hepatitis B (CHB), the hepatitis delta virus (HDV) is a viroid-like satellite that may co-infect people with HBV. Due to its flaws, HDV needs HBV structural proteins to produce virion. In CHB patients, the virus accelerates the development of liver disease to cirrhosis and raises the risk of HCC despite only encoding two versions of its one antigen. According to research, cells that overexpress the large HDV antigen (L-HDAg) or replicate the viral genome on their produce more ROS. Additionally, it results in the upregulation of the expression of the HCV-induced OS mediators’ cytochrome P450 2E1, ER oxidoreductase 1, nicotinamide adenine dinucleotide phosphate hydrogen (NADPH) oxidases 1 and 4, and NADPH oxidase 4. Additionally, the Nrf2/ARE pathway, which regulates the expression of various AOEs, was activated by both HDV antigens. Finally, ER stress and the associated unfolded protein response (UPR) were also triggered by HDV and its extensive antigen. As a result, HDV may exacerbate the oxidative and ER stress brought on by HBV, increasing the diseases connected to HBV, such as inflammation, liver fibrosis, and the development of cirrhosis and HCC [91] (Table 1) (Fig. 5).

**Table 1:** NRF2 function in hepatitis virus

Hepatitis virus	Explain study	Virus effects on NRF2	Result	Type of Study	Ref
HCV	During the early stages of HCV protein production in Huh7 cells, researchers have pinpointed the molecular mechanisms by which specific HCV proteins regulate the Nrf2/ARE pathway. These molecular processes may have a role in the acute phase of the illness when viral multiplication impacts the antioxidant defense system.	In addition to inducing high ROS levels, the core, E1, E2, NS4B, and NS5A proteins of HCV may also activate the Nrf2/ARE pathway. Multiple protein kinases are involved in the activation, which is only partly ROS-dependent.	Taking antioxidants during acute hepatitis C infection may not be the best idea. Patients should be treated with antioxidants to prevent the long-term consequences of stress because of the possibility that the Nrf2/ARE pathway will be inactivated during the chronic stage.	In vitro (HUH7 Cells)	[86]
HCV	Scientists want to learn more about how HCV’s atypical mechanism leading to an impeded Nrf2/ARE-signaling in HCV-replicating cells affects the activation of autophagy. Autophagy, viral particle release, increased ROS levels, pS349 p62 production, and reduced Nrf2/ARE-signaling are all interconnected processes that will be better understood.	HCV counteracts pS349 p62-induced activation of Nrf2 by suppressing Nrf2/ARE-mediated gene expression. This keeps ROS levels high, which triggers autophagy and promotes HCV particle release.	This makes the viral life cycle and virus-associated pathophysiology a potential target for restoring Nrf2/ARE signaling, which results in ROS detoxification.	In vitro (Huh7-derived cell line Huh7.5)	[87]
HBV	Studying the physiological importance of the viral life cycle and pathogenesis, researchers assess HBV’s ability to control the expression of ARE-regulated genes, uncover the processes by which HBV interferes, and more.	HBV-positive cells are better protected from oxidative damage because of the Nrf2/ARE-mediated activation of cytoprotective genes. In addition, the expression of the Nrf2/ARE-regulated proteasomal component PSMB5 is markedly upregulated in HBV-positive cells, whereas the expression of PSMB5i downregulated significantly.	The immune system and endoplasmic reticulum overload do not cause as much oxidative damage to HBV-positive cells. Keeping the Nrf2/ARE system functional is crucial for host tissue survival during liver regeneration. Increased constitutive proteasome activity guarantees that unfolded proteins are removed quickly and effectively. Reduced antigen processing occurs due to impaired immunoproteasome activity.	In vitro (HepG2, Huh7.5, and the HBV-positive stable cell lines HepG2.2.15 and HepAD38)	[84]
HBV	Scientists explain how HBV/G-infected cells cannot secrete HBsAg and identify elements that contribute to the pathogenesis of the virus.	An increased number of ROIs is caused by intracellular HBsAg buildup and poor Nrf2 cytoprotective transcription factor activation. The ability to inactivate ROIs declines along with a reduction in HBV-induced Nrf2 activation.	The reduced ability of HBV/G-expressing cells to inactivate ROIs is consistent with the reduced induction of Nrf2-dependent genes, which results in a reduced induction of cytoprotective mechanisms. This may have implications for understanding the pathogenesis of viruses.	In vitro (HuH7.5 and HepG2)	[90]
HBV	This research aimed to characterize mechanisms leading to HBV/ G-associated pathogenesis by contrasting the morphogenesis and release of HBV-derived particles between HBV genotypes G and A2.	Genotype G’s aggregating PreS1PreS2 domain prevents ER access for HBsAg secretion and reduces the transcriptional activator activity of LHBs. Increased levels of ROIs are caused by the intracellular buildup of HBsAg and the failure to induce the cytoprotective transcription factor Nrf2. As a result, Ser-phosphorylation of IRS-1 occurs, which is known to disrupt insulin signaling and impede liver regeneration.	HBV/G infection, on the other hand, inhibits the Nrf2 pathway because of the accumulation of subviral HBsAg particles within the cell, and the expression of Nrf2 target genes is reduced in HBV/G replicating cells. In conclusion, Keap1/Nrf2 signaling is modulated in various ways by HBV genotype-specific regulatory proteins, which in turn uniquely affects the virus’s pathogenicity.	In vitro (HuH7.5 and HepG2)	[20, 90]
HDV	The purpose of this investigation was to examine how HDV antigens affect ROS generation and the expression of several ROS-generating enzymes, all of which have been reported in the context of other hepatitis viruses. Second, researchers wanted to see whether we could find evidence of a problem with the antioxidant defense Nrf2/ARE pathway. The third objective was to research how HDV antigens affect ER stress and the subsequent UPR. In this study, the authors demonstrated that L-HDAg, in conjunction with S-HDAg, promotes the ROS-producing enzymes NOX1, NOX4, CYP2E1, and Ero1, activates the Nrf2/ARE defensive pathway and triggers the UPR.	The Nrf2/ARE pathway, which regulates the expression of various AOEs, was activated by both HDV antigens. Finally, ER stress and the associated UPR were also triggered by HDV and its prominent antigen. As a result, HDV may exacerbate the oxidative and ER stress brought on by HBV, increasing the diseases connected to HBV, such as inflammation, liver fibrosis, and the development of cirrhosis and HCC.	Overexpression of a series of ROS-generating extramitochondrial enzymes, activation of the Nrf2/ARE pathway that controls the expression of antioxidant enzymes, and induction of ER stress and a concomitant unfolded protein response are all triggered by HDV replication in liver cell lines and the simultaneous expression of the viral antigen(s). The importance of these occurrences in HDV pathogenesis may be shown in future research.	In vitro (Huh7 cells)	[91]

## NRF2 in HBV and HCV-related liver cancer

**Fig. 6:**
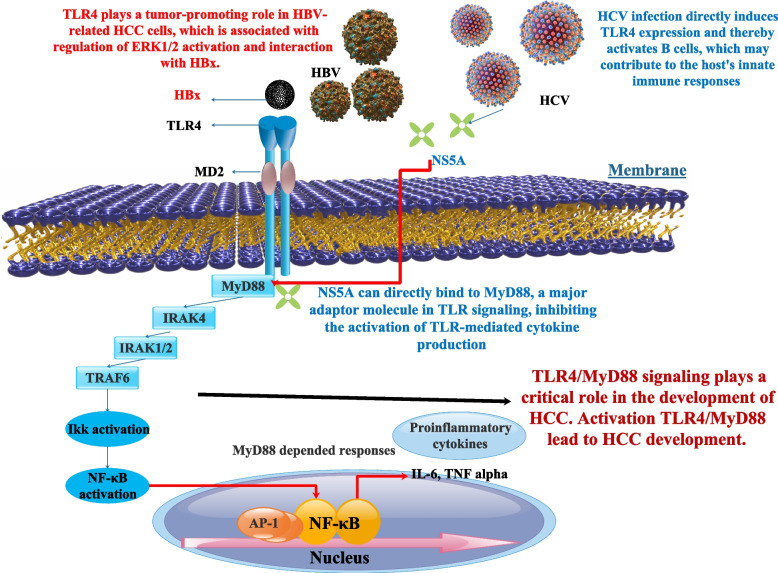
To melanoma, TLR4 is linked to metastasis in other cancers because its activation causes cell migration. TLR signaling is upregulated in HCC, which implies it may play a crucial role in the prognosis of the underlying chronic and inflammatory disorders that lead to HCC [99]. Activation of B cells, which may play a role in the host's innate immune responses, is a direct result of HCV infection's induction of TLR4 expression. Nonstructural Protein 5A (NS5A) of varying genotypes linked to MyD88, a crucial adaptor protein in TLR, and reduced cytokine release in response to TLR ligands by blocking the recruitment of interleukin-1 receptor-associated kinase 1. The development of a mutant NS5A missing the interferon sensitivity-determining region (ISDR) partly restored cytokine production. The ISDR was previously identified as amino acid residues 240 to 280 in NS5A. These findings point to a connection between HCV protein expression and alterations in immune cell TLR signaling [100]. Activation of ERK1/2 and contact with HBx are two mechanisms by which TLR4 promotes tumorigenesis in HBV-related HCC cells [101]

HBV-induced liver disorders are exacerbated by interleukin (IL)-17 A. It has only been recently discovered that IL-17 and its byproducts play crucial roles in the development of chronic HBV-related liver disorders. Significantly elevated IL-17 A and IgE levels were connected to the IL17A rs2275913 HBV/G variant. Polymorphisms in IL17A, which control IL-17 A production, may affect hepatocellular carcinoma risk in patients with persistent HBV infection [92, 93]. Few of these studies contain a leukocyte recruitment phenotype, whereas most attribute IL-17’s role to its production in adaptive Th17 cells and subsequent activation of cytokine release [94]. Both the mRNA and protein levels of IL-17 A and IL-23 expression were increased in response to TLR4/MyD88 activation. TLR4 expression was also increased after MyD88 activation. TNM stage and tumor metastasis seemed to be linked with IL-17 A and IL-23 expression levels in HCC. As a result of its modulation of the IL-23/IL-17 A axis, the TLR4/MyD88 signaling pathway contributes to HCC cell proliferation and metastasis [95] (Fig. 6). In addition, the serum levels of IL-17 increased significantly in those patients suffering from chronic HCV and HCC [96]. Scientists demonstrated that IL-17D, a cytokine, influences extrinsic stress surveillance by eliciting an immune response to tumors. Further, IL-17D was activated by Nrf2 in cancer cell lines. Not only was Nrf2 activated in primary tumors, but IL-17D was also following viral infection in vivo. Due to a greater tumor incidence and worsened viral infections in il17d-/- mice compared to wild-type (WT) animals, it is clear that IL-17D expression in tumors and virally infected cells is crucial for optimum protection of the host. Additionally, activating Nrf2 to cause IL-17D in pre-existing tumors resulted in tumor regression mediated by natural killer cells. Nrf2 and IL-17D are produced by viral infection, most likely as a result of local OS. Since animals lacking in IL-17D showed increased development of MCA-induced tumors and worsened pathology when infected with vaccinia virus (VV) or murine cytomegalovirus (MCMV), IL-17D production is necessary for efficient cancer surveillance and antiviral responses. These findings emphasize the utility of Nrf2 agonists as immunological treatments for cancer and infection and show that Nrf2 may start internal and extrinsic stress surveillance mechanisms. By inducing local OS, HBV infection probably triggers Nrf2 and IL-17D. Additionally, HBV may stimulate Nrf2 to cause IL-17D in existing tumors [97]. By activating the transcription factor Nrf2, phosphorylation of p62/Sqstm1 at Ser349 drives glucose to the glucuronate pathway and glutamine to glutathione production. These modifications provide HCC cells with the ability to tolerate anti-cancer medications and to proliferate. Amounts of phosphorylated p62/Sqstm1 build up in HCV-positive tumor areas. HCC’s ability to proliferate and withstand treatment with anticancer agents is suppressed by an inhibitor of phosphorylated p62-dependent Nrf2 activation. According to this study’s findings, this Nrf2 inhibitor may reduce cancer cells’ resistance to anticancer medications, particularly in HCC patients who also have HCV [98].

An essential controller of the OS response is glycogen synthase kinase (GSK) 3β. Hepatocytes exposed to HCV infection exhibited a strong Nrf2 antioxidant response, as shown by increased production of the Nrf2-dependent molecule hemoglobin oxygenase-1 and protection against apoptotic cell death. For the HCV-induced Nrf2 response, inhibitory phosphorylation of GSK3β seems necessary and sufficient. GSK3β mechanically connected to and interacted with Nrf2 in hepatocytes. In-silico research showed that Nrf2 has various GSK3β phosphorylation consensus patterns, designating Nrf2 as a GSK3β-specific substrate. TGFβ1 significantly reduced the cytoprotective Nrf2 response and inhibited the HCV-induced GSK3β phosphorylation via a protein phosphatase 1-dependent mechanism. Lithium, a GSK3β-selective inhibitor, blocked this effect. The level of phosphorylated GSK3 was negatively connected with the severity of liver damage and positively correlated with Nrf2 expression in liver biopsy samples from individuals with combined hepatocellular and cholangiocarcinoma (CHC). Additionally, individuals with CHC who had long-term lithium carbonate medication primarily for coexisting mental problems showed much-reduced liver damage, which was connected to increased Nrf2 hepatic expression [102]. Researchers have shown that CHB infection of the human liver and HBV-related liver cancer is linked with high expression of glucose-6-phosphate dehydrogenase (G6PD), the first and rate-limiting enzyme of the pentose phosphate pathway, as well as increased Nrf2 transcription factor activity. HBx increases the production of G6PD in hepatocytes via a mechanism reliant on Nrf2 activation. To establish the HBx-p62-Keap1 complex in the cytoplasm, HBx interacts with the ubiquitin-associated (UBA) and Phox and Bem1(PB1) domains of the adaptor protein p62 and enhances its connection with the Nrf2 repressor Keap1. Keap1 is taken over by HBx-p62-Keap1 complexes, which activate Nrf2 and subsequently cause G6PD transcription. These findings imply that HBV increases G6PD expression via the activation of Nrf2 by HBx. This suggests that HBV may impact the reprogramming of glucose metabolism in hepatocytes, which may be significant in the emergence of hepatocarcinoma linked with HBV [103].

Six hundred seventy-three participants were divided into four groups: 217 healthy controls, 260 HCC cases, 260 liver cancer (LC) cases, and 110 CHB cases. The rs6721961 and rs6726395 polymorphisms were discovered using DNA sequencing and the polymerase chain reaction-restriction fragment length polymorphism technique. Compared to CHB patients, those with the T allele in rs6721961 had a greater risk of developing HCC (OR = 1.561, 95%CI: 1.003–2.430, *P* = 0.048). The rs6721961 GT genotype (OR = 2.298, 95% CI: 1.282–4.119, *P* = 0.005) and dominant model (OR = 2.039, 95% CI: 1.184–0.510, *P* = 0.010) also revealed statistically significant differences. A substantial connection between the rs6721961 T allele and the emergence of HCC in older individuals (> 50 years) was also discovered by subgroup analysis (OR = 2.148, 95% CI: 1.208–3.818, *P* = 0.009). Results of the statistical analysis showed that individuals with the haplotype G-A had a decreased chance of developing HCC (OR = 0.700, 95% CI: 0.508–0.965, *P* = 0.028). These findings indicate the potential significance of the rs6721961 polymorphism in HCC susceptibility and the development of this illness. They are the first to our knowledge to quantify genetic polymorphisms of NRF2 in the risk of CHB, HBV-related liver cirrhosis, and HCC [104].

Protecting the liver from various assaults relies heavily on the KEAP1-Nrf2 antioxidant signaling pathway. Liver tissues from healthy donors (*n* = 35), patients with hepatocellular carcinoma (HCC; *n* = 24), those with hepatitis B virus-related cirrhosis (*n* = 27), those with alcoholic cirrhosis (*n* = 5), and those with end-stage liver disease (*n* = 13) were used in this investigation. The liver tissue used in this study was obtained from the Oriental Liver Transplant Center in Beijing, China. In this study, Researchers analyzed the expression levels of Nrf2 and Nrf2-related genes such as NAD(P)H-quinone oxidoreductase 1 (NQO1), glutamate-cysteine ligase catalytic subunit (GCLC), modified subunit (GCLM), HO-1, and peroxiredoxin-1 (PRDX1). Compared to alcoholic cirrhosis and advanced liver disease, HCC was associated with a reduction in Nrf2 expression. Increased KEAP1 expression was seen across the board in the liver tissue samples. The most striking observation was that NQO1 expression was significantly upregulated in a wide variety of liver diseases, including HCC (18-fold), alcoholic cirrhosis (6-fold), end-stage liver disease (5-fold), and HBV-related cirrhosis (3-fold). NQO1 mRNA was found to be four times greater in peri-HCC than in normal livers. Only in HCC were glutamate-cysteine ligase catalytic (GCLC) mRNA levels lower than in normal livers and peri-HCC tissues. Cirrhosis and liver failure caused by HBV were associated with increased levels of GCLM mRNA. All liver tissues, except for HCC, had elevated HO-1 mRNA levels. In comparison to HCC and normal livers, Peroxiredoxin 1 (PRDX1) mRNA levels were significantly greater in peri-HCC [105].

## Conclusion

Due to its many functions, including antioxidant, metabolic, and anti-inflammatory, Nrf2 is an essential participant in the systems that control cell transformation and the immune response to viral infections. It is difficult to assign a positive or negative value to a component like Nrf2 in the outcome of a disease like cancer due to the molecular complexity that defines cancer genesis and progression. However, when Nrf2 is over-stimulated or over-expressed, it promotes OS and inflammation associated with carcinogenesis; hence it must be considered an oncogenic factor. Understanding the molecular basis of cancer and using targeted anti-tumor medicines that either stimulate or inhibit Nrf2 may require being able to distinguish such behavior under various circumstances [106]. Overall, Nrf2 has both pro- and anti-tumor effects, including, for example, fostering cell growth and treatment resistance. Due to its essential function, Nrf2 is now being studied as a potential cancer-fighting drug target. To create medications with high specificity and low adverse effects, however, a better understanding of the molecular mechanism leading to tumor suppression or the oncogenic action of Nrf2 is required [107]. As a result, it would be ideal to create a new agent with these dual properties that could be used alone or in conjunction with current anticancer treatments. NRF2 has therapeutic promise for treating viral infectious diseases because it may play a key role in antiviral immunity. It is crucial to establish a connection between liver disorders and OS. By regulating protective genes, the Nrf2 antioxidant system shields the liver from damage; this shield extends to cells affected with viral hepatitis and HCC. It has also been shown that HCV activates Nrf2 and that HCCs with the virus present (HCV-positive HCC, or C-HCC) have enhanced p62/Sqstm1 pathways, which promotes metabolic reprogramming and tumor growth in a manner that is reliant on Nrf2. The conflicting findings regarding Nrf2 activation status in HCV infection may be result from varying experimental setups and settings. Additionally, the Keap1/Nrf2 signaling pathway and the corresponding virus-associated pathogenetic processes are also affected differently by the regulatory proteins of the various HBV genotypes. Numerous preclinical studies have found Nrf2 regulatory factors; however, further research has revealed Nrf2 activators for liver injury/failure and Nrf2 inhibitors for viral hepatitis, HCC, and both. This research is encouraging because it could lead to the development of comprehensive and efficient methods to improve the prognosis of liver diseases. Therefore, the focus of future studies will be on how NRF2 hyperactivity contributes to the development of tumors and medication resistance. To further understand the underlying processes and explore how NRF2 mutations contribute to the emergence of liver cancer, more research is required.

## Data Availability

Not applicable.
